# Supplementary material for: Comprehensive innate immune profiling of chikungunya virus infection in pediatric cases
Source: Mol Syst Biol. 2018 Aug 27;14(8):e7862. doi: 10.15252/msb.20177862 (PMC6110311; doi:10.15252/msb.20177862)
Supplement: Supplementary file 1 — Appendix [file MSB-14-e7862-s001.pdf]

Appendix

Comprehensive innate immune profiling of chikungunya virus infection in pediatric cases

Authors

Daniela Michlmayr,*[1] Theodore R. Pak,*[2] Adeeb H. Rahman,[2,3] El-Ad David Amir,[2,3] Eun-Young Kim,[4] Seunghee Kim-Schulze,[2,3] Maria Suprun,[5] Michael G. Stewart,[4] Guajira P. Thomas,[4] Angel Balmaseda,[6] Li Wang,[2] Jun Zhu,[2] Mayte Suarez-Fariñas,[2,5] Steven M. Wolinsky,[4] Andrew Kasarskis,[2] and Eva Harris[1#]

* These authors contributed equally to this work.

# Corresponding author: Eva Harris, PhD; Division of Infectious Diseases and Vaccinology, School of Public Health, University of California, Berkeley, 185 Li Ka Shing Center, 1951 Oxford Street, Berkeley, CA 94720-3370; email: eharris@berkeley.edu; phone: (510) 642-4845

[1] Division of Infectious Diseases and Vaccinology, School of Public Health, University of California, Berkeley, CA, 94720, USA

[2] Department of Genetics and Genomic Sciences, Icahn School of Medicine at Mount Sinai, New York, NY, 10029, USA

[3] Human Immune Monitoring Center, Icahn School of Medicine at Mount Sinai, New York, NY, 10029, USA

[4] Division of Infectious Diseases, Department of Medicine, Northwestern University Feinberg School of Medicine, Chicago, IL, 60611, USA

[5] Department of Population Health and Science Policy, Icahn School of Medicine at Mount Sinai, New York, NY, 10029, USA

[6] Laboratorio Nacional de Virología, Centro Nacional de Diagnóstico y Referencia, Ministerio de Salud, Managua, Nicaragua

Appendix Table of Contents

Appendix Figures..3

Appendix Figure S1................................4
Appendix Figure S2................................5
Appendix Figure S3................................6
Appendix Figure S4................................7
Appendix Figure S5................................8
Appendix Figure S6..............................10
Appendix Figure S7..............................11
Appendix Figure S8..............................12
Appendix Figure S9..............................13
Appendix Figure S10............................14
Appendix Figure S11............................15
Appendix Figure S12............................16
Appendix Figure S13............................17
Appendix Figure S14............................18
Appendix Figure S16............................20
Appendix Figure S17............................21
Appendix Figure S18............................22
Appendix Figure S19............................23
Appendix Figure S20............................24
Appendix Figure S21............................25
Appendix Figure S22............................26
Appendix Figure S23............................27
Appendix Figure S24............................28
Appendix Figure S25............................29
Appendix Figure S26............................30
Appendix Figure S27............................31
Appendix Figure S28............................32
Appendix Figure S29............................33
Appendix Figure S30............................34

Appendix Figures

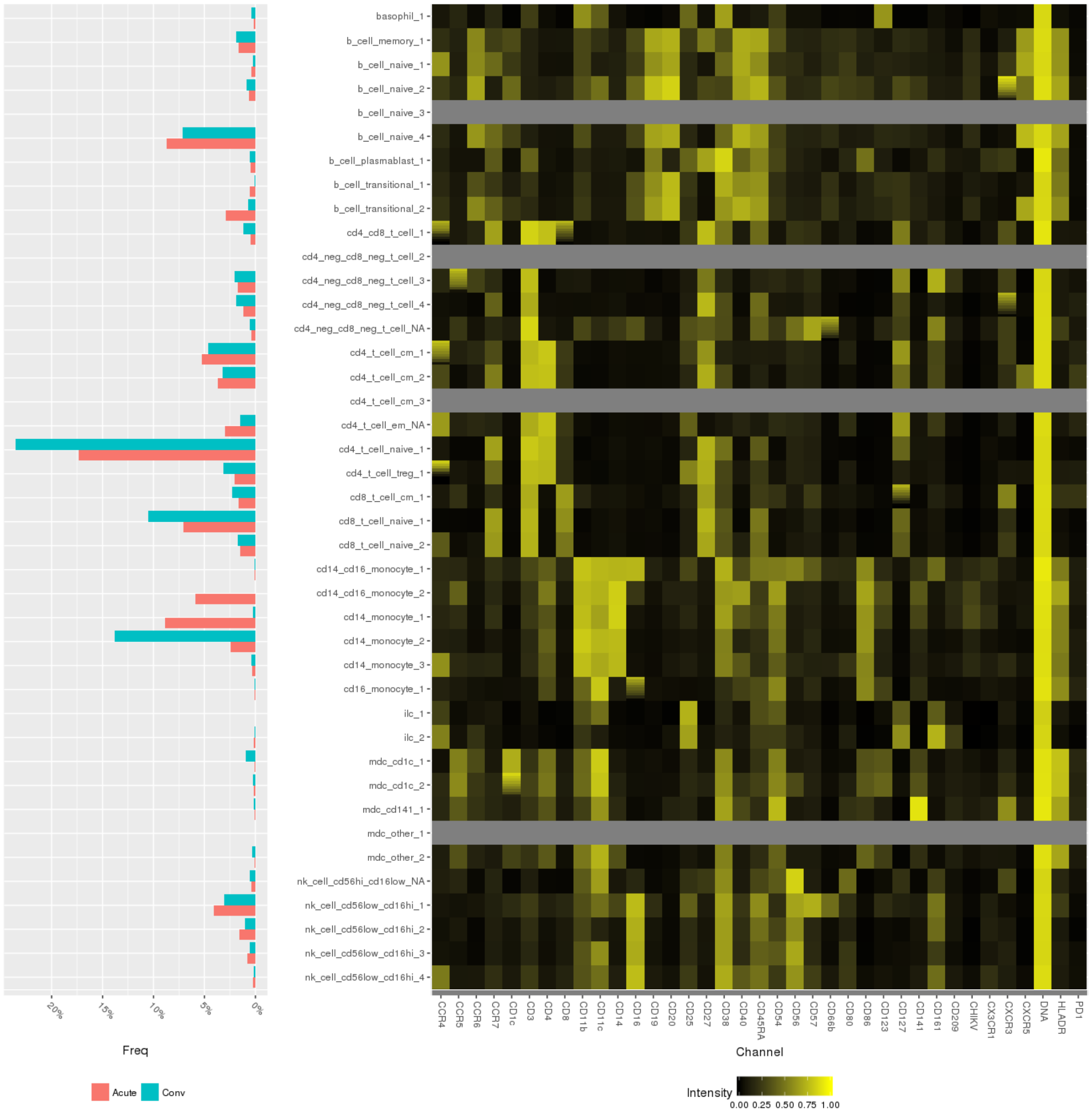

continued on next page

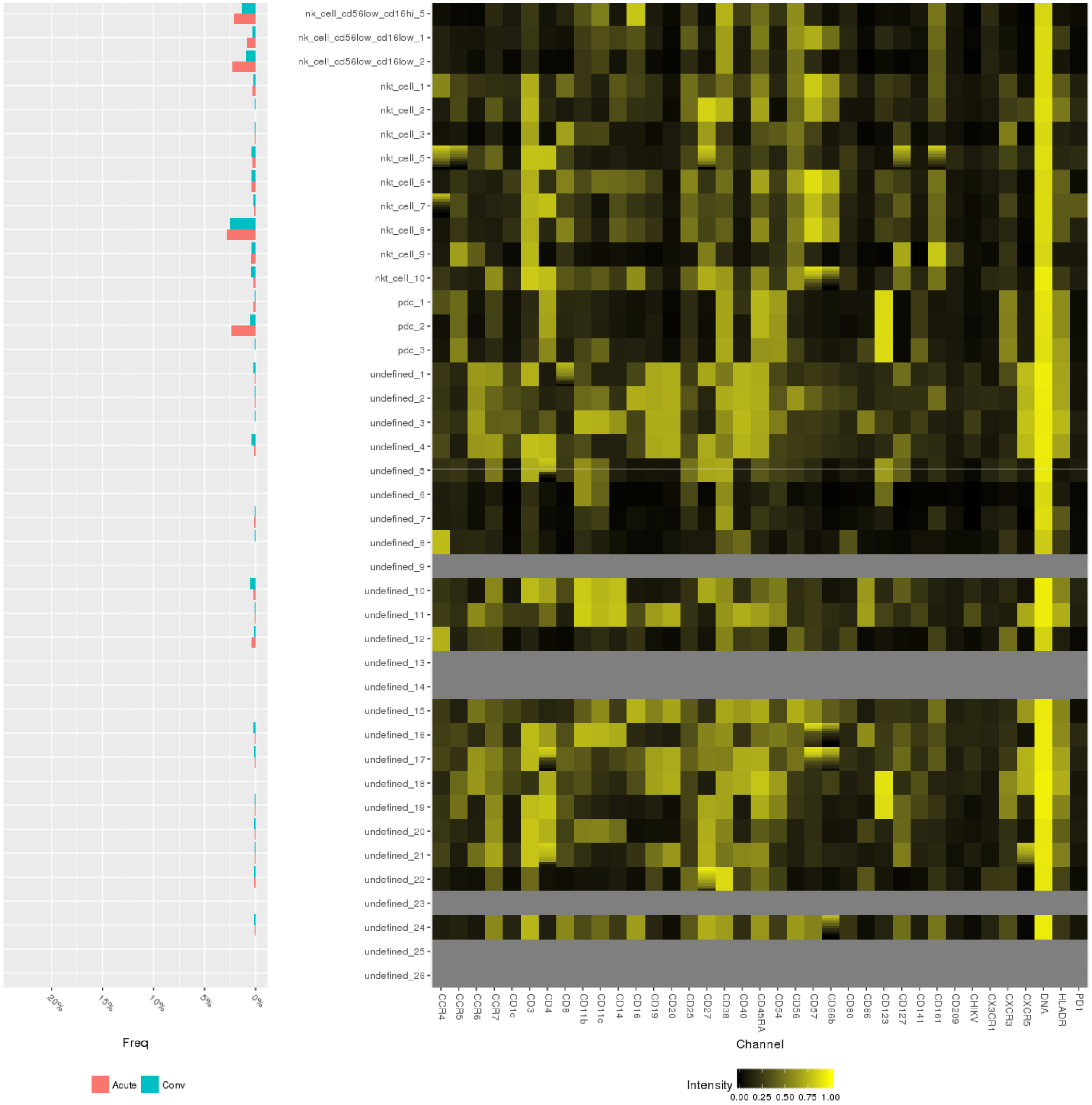

Appendix Figure S1.

Example output of MetaHybridLouvain for the representative sample used in Figure 2A, Figure 3B, and 3D. At left, frequencies for each sub-community at each timepoint, and at right, a heatmap of the distribution of channel values is plotted for each sub-community (within each row). As expected, sub-communities generally show similar values among all channels for their constituent events, with some exceptions (heatmap cells with vertical gradients).

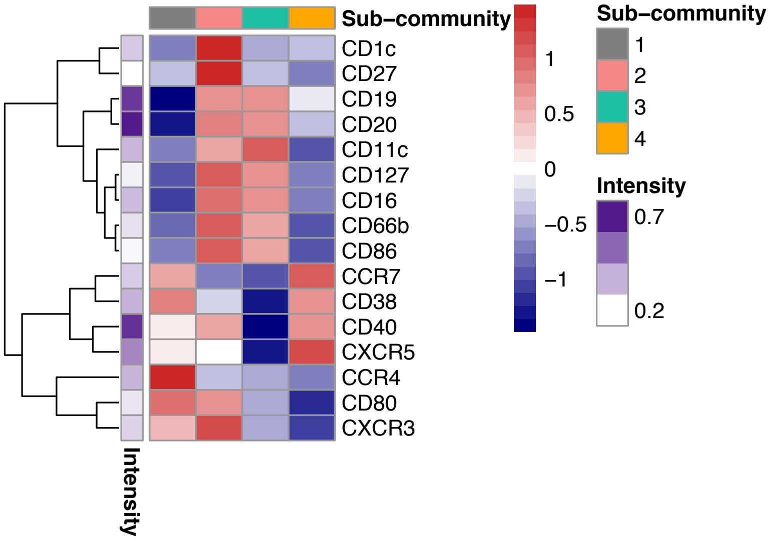

Appendix Figure S2.

Heatmap of differences in marker protein expression between four naïve B cell sub-communities identified by MetaHybridLouvain. All channels listed here differentiate sub-community 4 from the median value for the other three sub-communities at a threshold of fold change > 1.5 and FDR < 0.05 (using a moderated paired t-test under the mixed effects model). Colors represent $\log_2$ fold change in channel intensity from the median for all communities (red means higher intensity). Notably, sub-community 4 is characterized by a much higher expression of CXCR5 as compared to all other sub-communities of naïve B cells. Otherwise, its marker expression is very similar to sub-community 1 of naïve B cells, albeit with much lower expression of CCR4, CXCR3 and CD80.

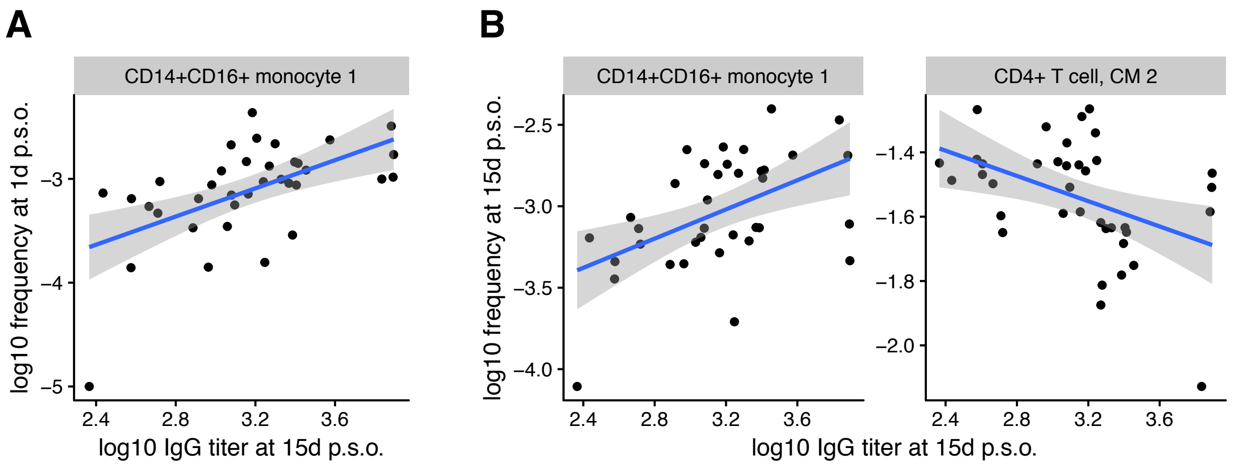

Appendix Figure S3.

Correlations between acute-phase cell sub-community frequencies and $\log_{10}$ CHIKV antibody titer at 15d post-symptom onset (p.s.o.). *A*, Scatterplot of $\log_{10}$ cell sub-community frequencies at 1d p.s.o. against $\log_{10}$ CHIKV antibody titer at the 15d timepoint for $CD14^{+}CD16^{+}$ monocyte sub-community 1. This is the only correlation that is significant after multiple hypothesis correction (FDR $P = 0.0050$, Spearman's $\rho = 0.60$). *B*, Scatterplot as in *A* but for two sub-communities that have significant correlations at the 15d p.s.o. timepoint: again, $CD14^{+}CD16^{+}$ monocyte sub-community 1 (FDR $P = 0.035$, $\rho = 0.51$), and central memory $CD4^{+}$ T cell sub-community 2 (FDR $P = 0.035$, $\rho = -0.52$).

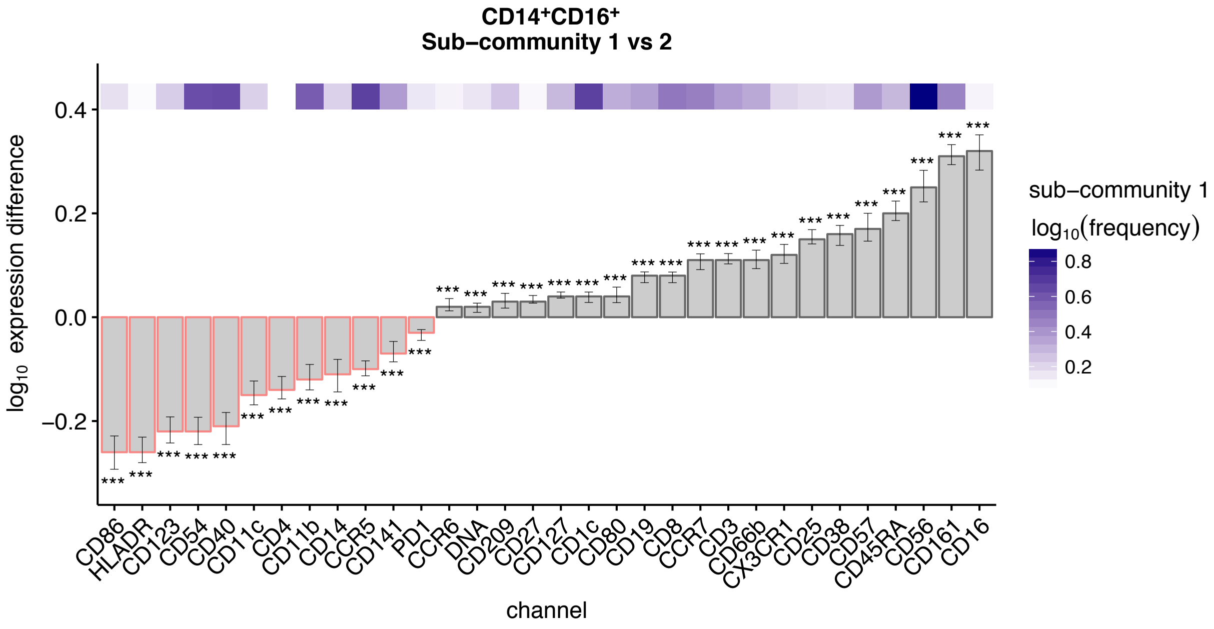

Appendix Figure S4.

Differences in per-sample protein expression between two $CD14^{+}CD16^{+}$ sub-communities identified by MetaHybridLouvain on CyTOF data. 1 corresponds with the “intermediate” $CD14^{++}CD16^{+}$ phenotype, while 2 corresponds with the “nonclassical” $CD14^{+}CD16^{++}$ phenotype. The X axis is filtered to channels with differences significant at FDR < 0.05, and ordered from differences where sub-community 1 > 2 on the left to sub-community 1 < 2 on the right; i.e., red bars are “intermediate”-associated markers while gray bars are “nonclassical”-associated markers. Error bars correspond to 95% confidence intervals. *, FDR-adjusted *P* (FDR *P*) < 0.05; **, FDR *P* < 0.01, ***, FDR *P* < 0.001.

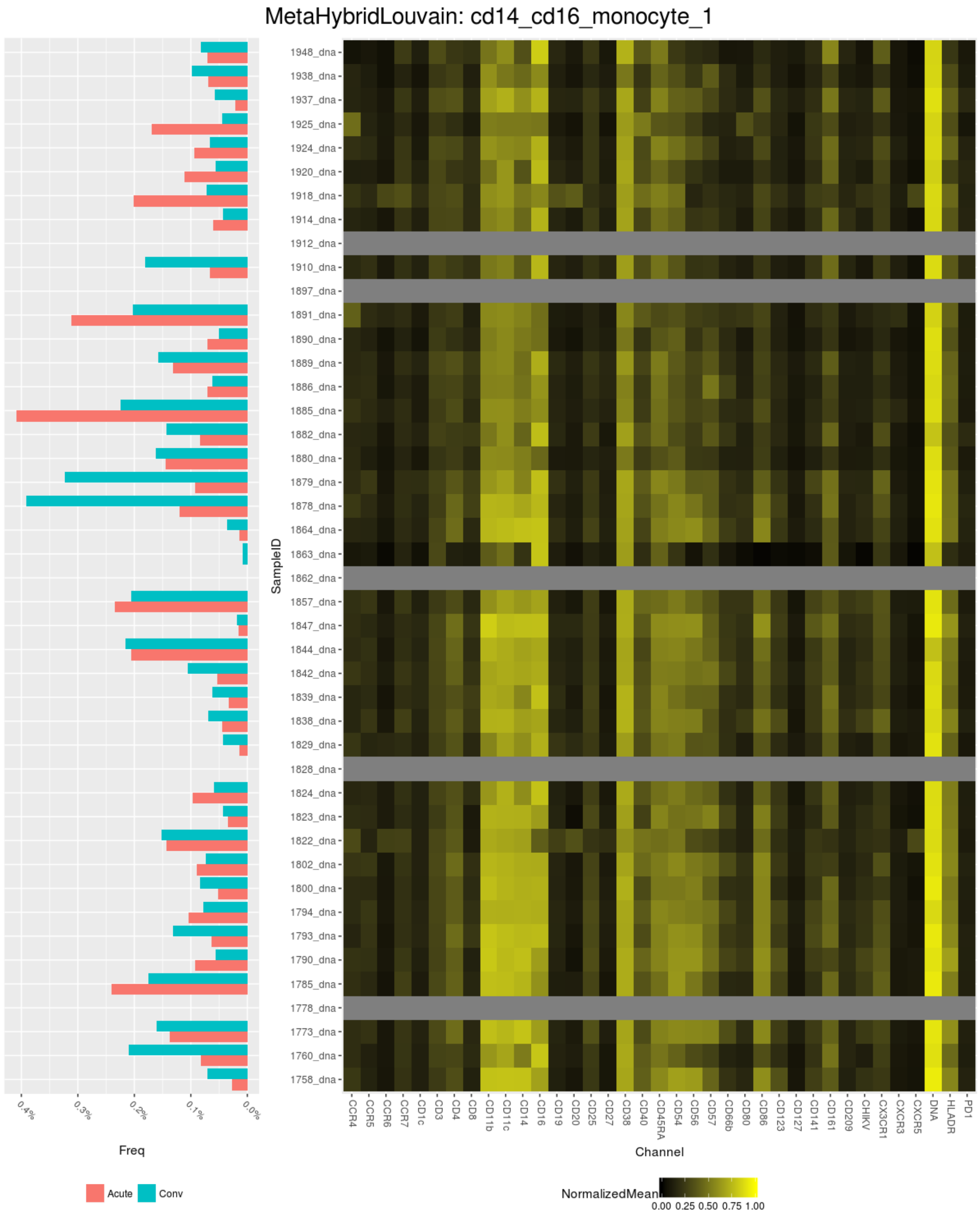

Appendix Figure S5.

Summary of frequencies (per timepoint) and mean channel values for sub-community 1 of $CD14^{+}CD16^{+}$ monocytes, across all samples. At left, frequencies in each sample split by

timepoint; at right, within each row, heatmaps of the distribution of channel values for all events within this sub-community for each sample. A gray row indicates this sub-community was not identified in this sample.

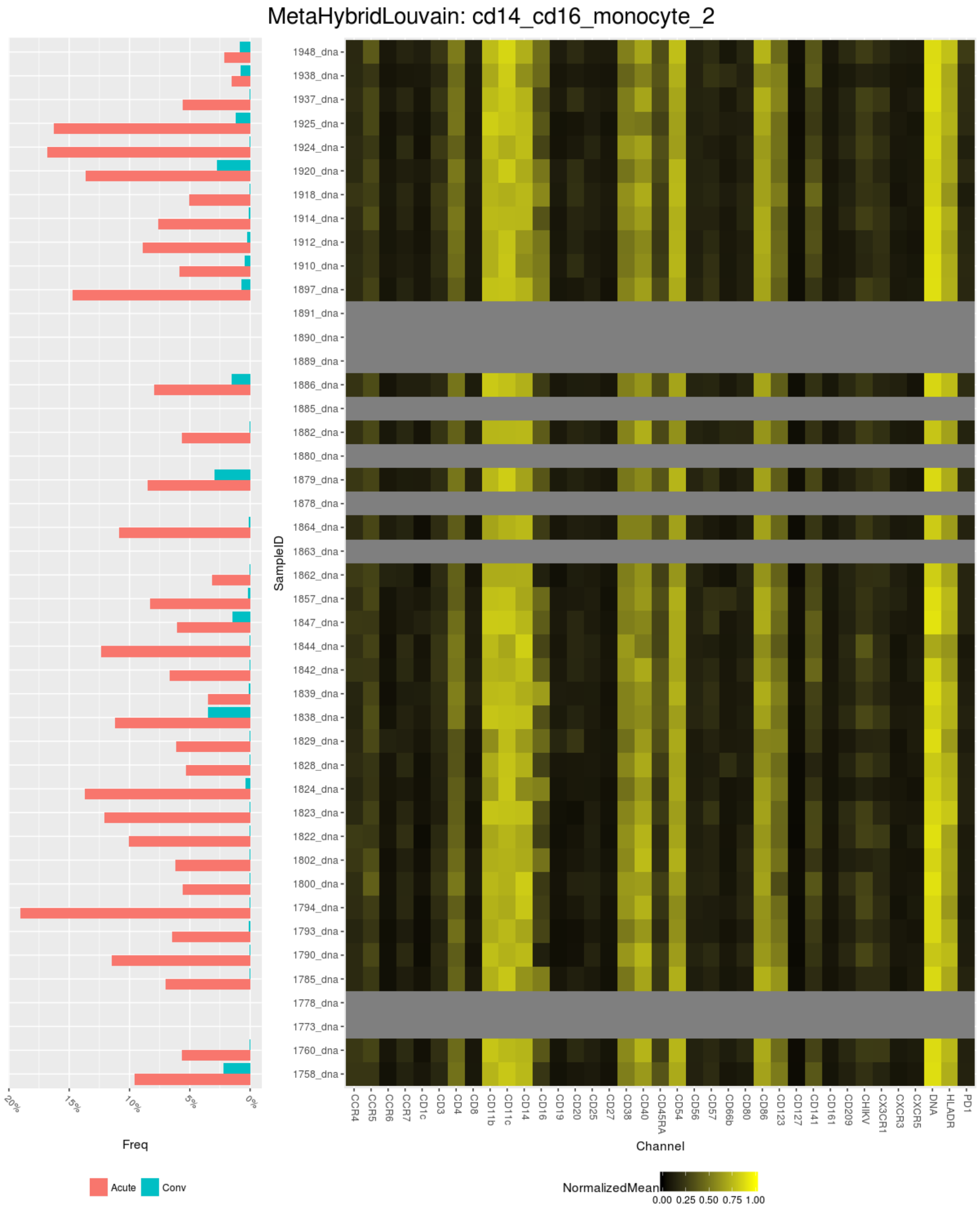

Appendix Figure S6.

Summary of population frequencies (per timepoint) and mean channel values for sub-community 2 of CD14^{+}CD16^{+} monocytes, across all samples. Layout as in Appendix Figure S5.

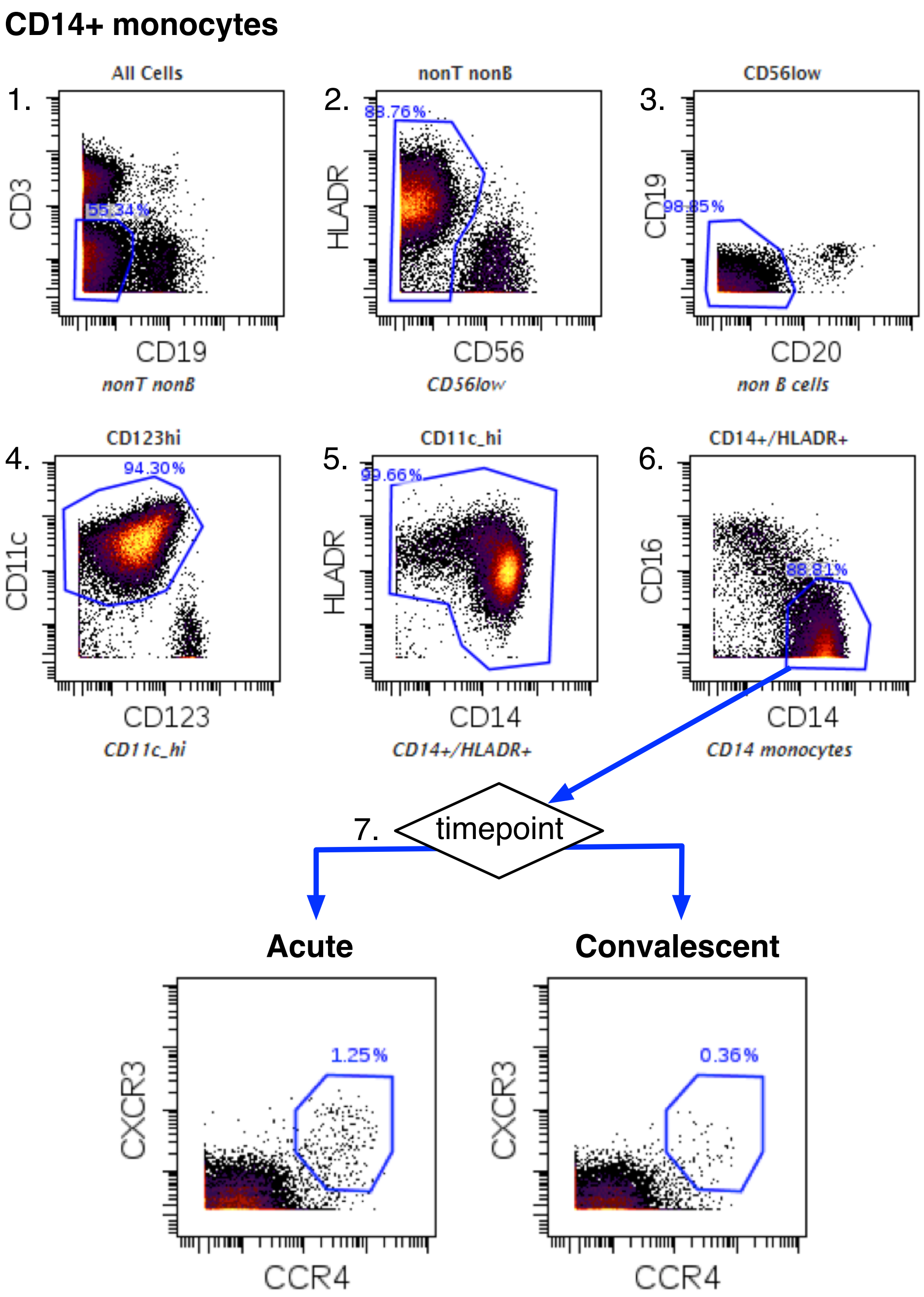

Appendix Figure S7.

Manual gating analysis of a representative sample demonstrates the presence of a small but distinct CD14^{+} monocyte subpopulation that displays non-canonical markers CXCR3 and CCR4, among others (see sub-community 3 in Appendix Figure S8).

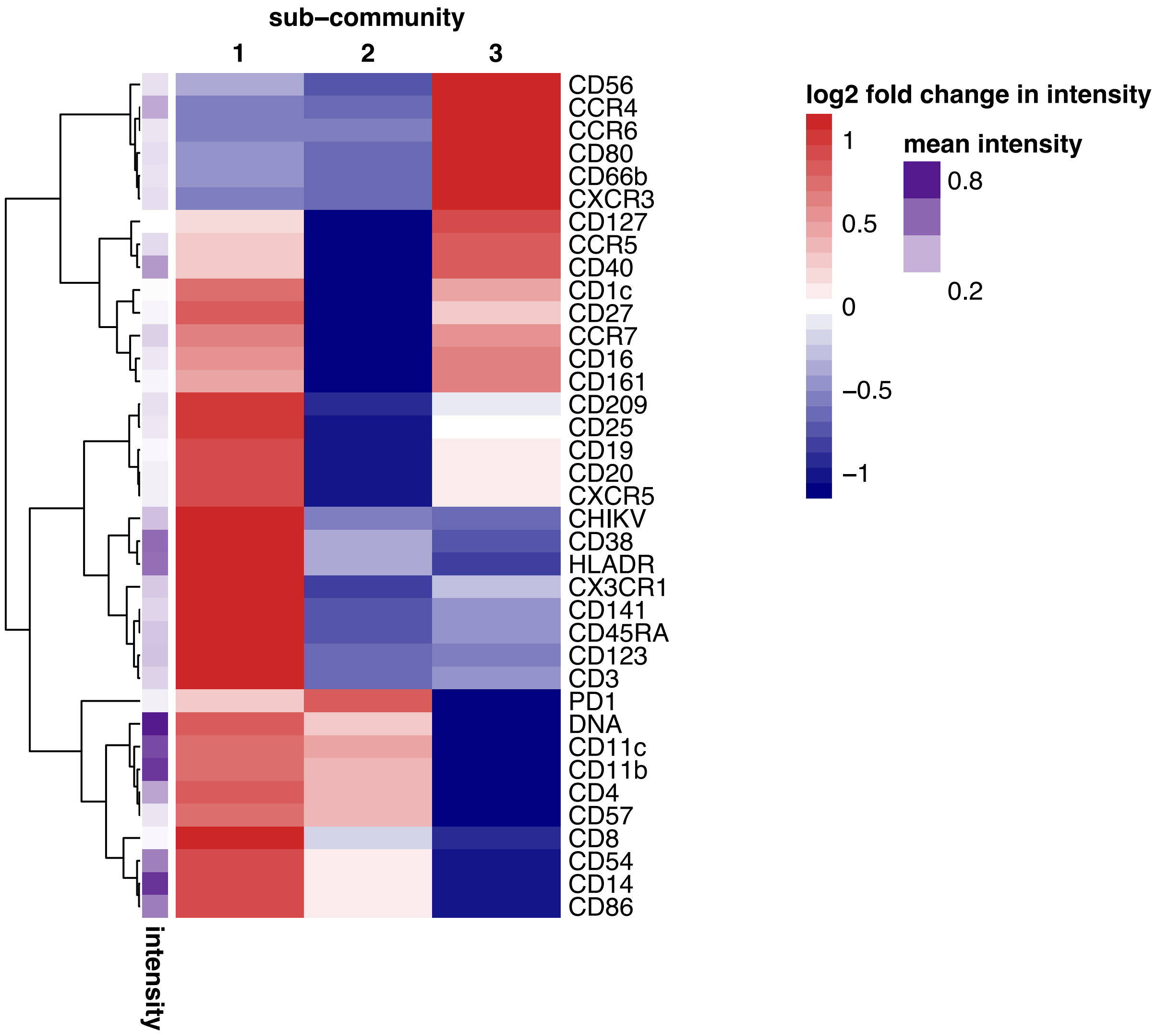

Appendix Figure S8.

Heatmap of differences in marker protein expression between three $CD14^{+}$ sub-communities identified by MetaHybridLouvain. All channels listed here showed differences significant at FDR < 0.05 (moderated paired t-test under the mixed-effects model). The patterns of fold change differences in channel intensities between sub-communities are ordered vertically with hierarchical clustering (red indicates increased relative to the other sub-communities). The mean intensity of each channel across *all* sub-communities is shown by the adjacent purple annotation column.

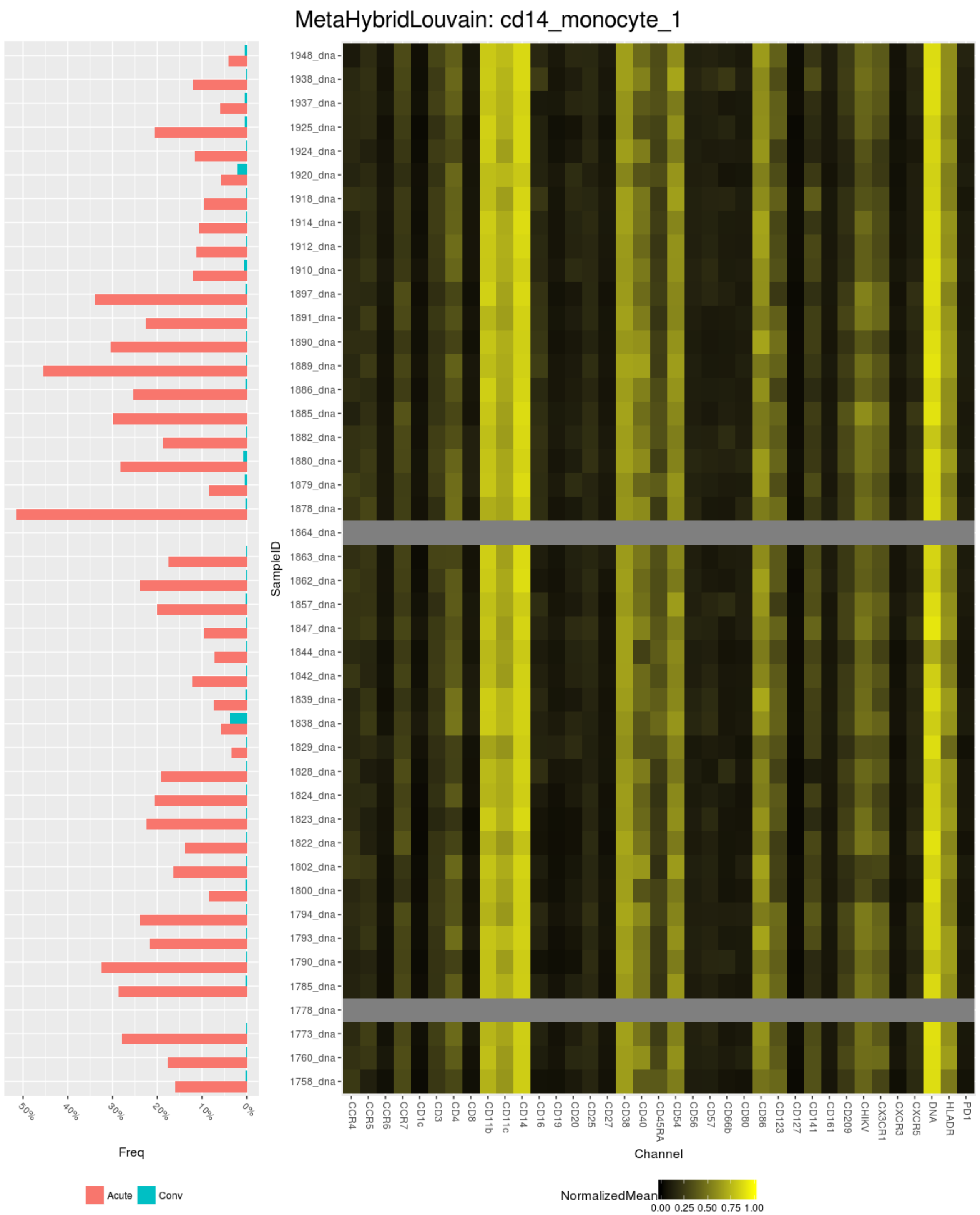

Appendix Figure S9.

Summary of population frequencies (per timepoint) and mean channel values for sub-community 1 of $CD14^+$ monocytes, across all samples. Layout as in Appendix Figure S5.

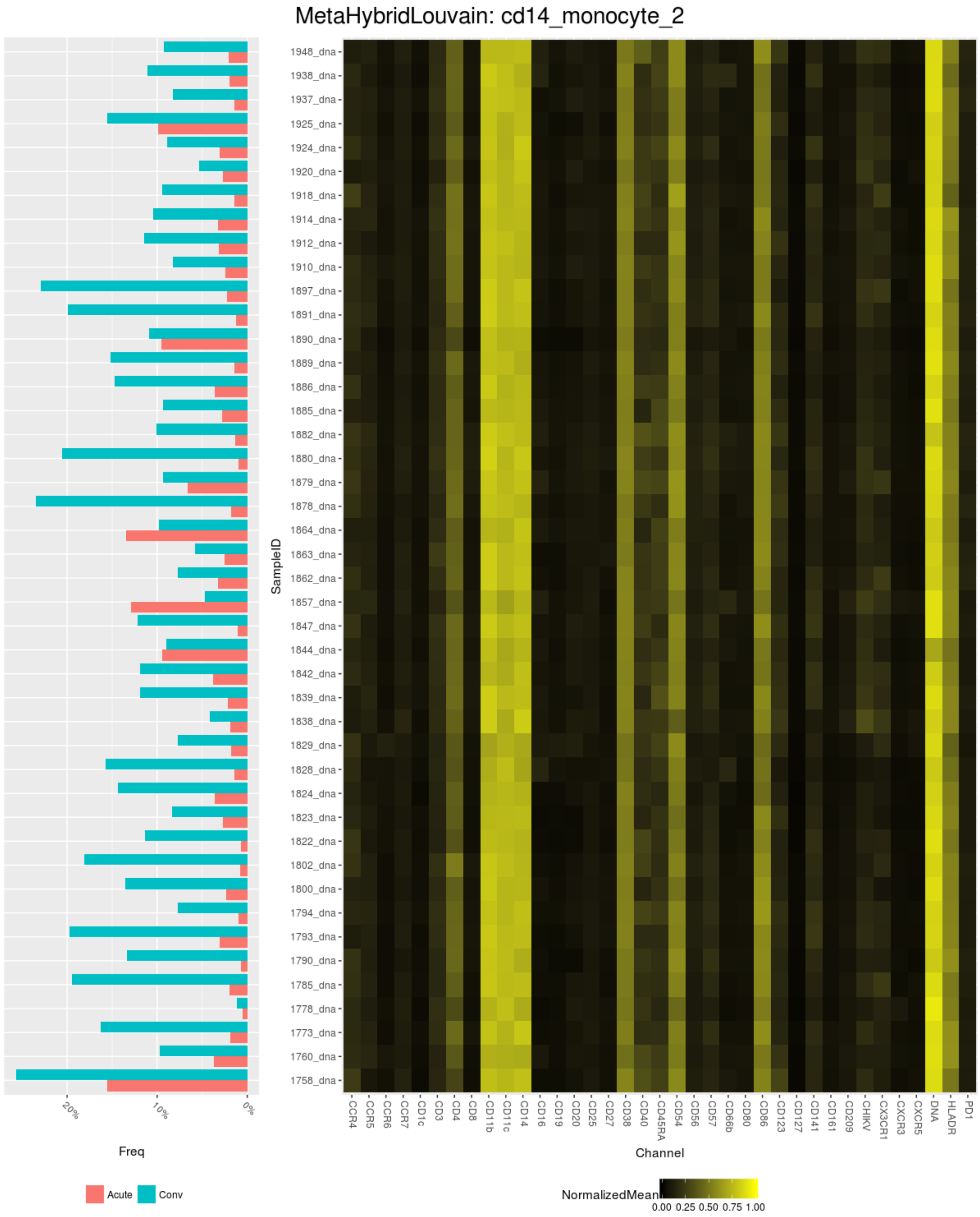

Appendix Figure S10.

Summary of population frequencies (per timepoint) and mean channel values for sub-community 2 of CD14^{+} monocytes, across all samples. Layout as in Appendix Figure S5.

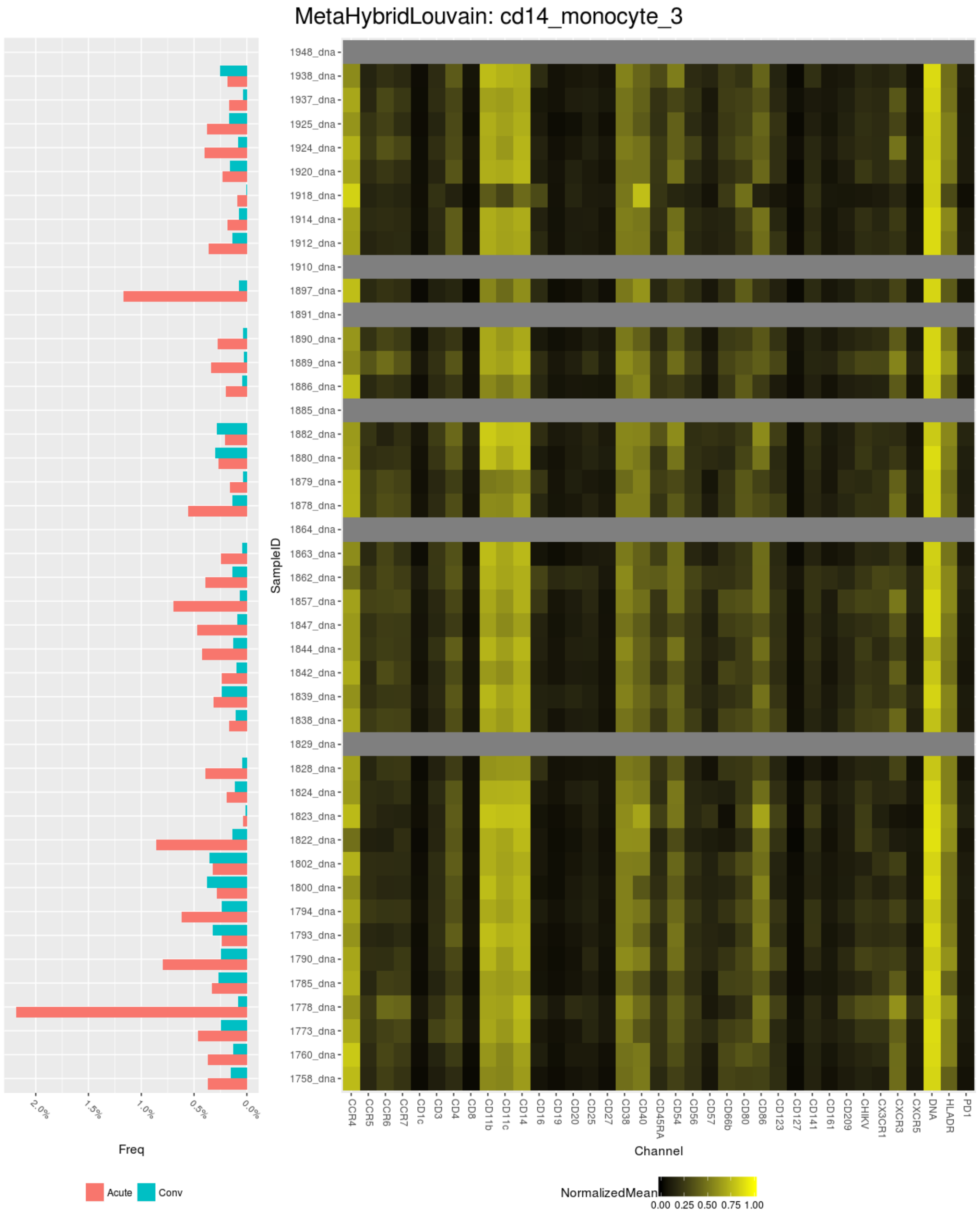

Appendix Figure S11.

Summary of population frequencies (per timepoint) and mean channel values for sub-community 3 of CD14^{+} monocytes, across all samples. Layout as in Appendix Figure S5.

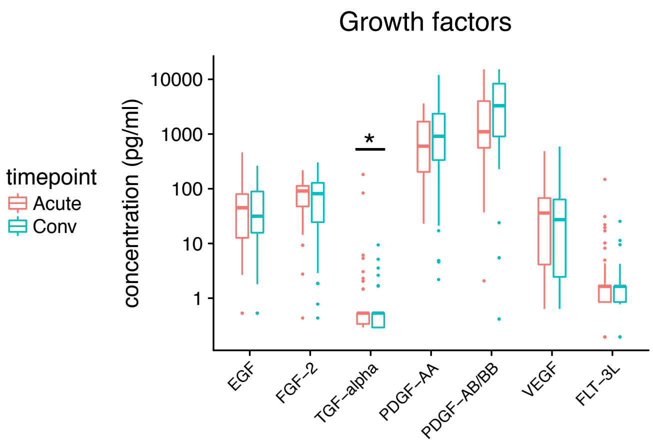

Appendix Figure S12.

Differences in serum growth factor levels between the acute and convalescent timepoints, as measured by multiplex ELISA (Luminex). Only one of the differences depicted here achieved statistical significance at FDR < 0.05, indicated by the asterisk.

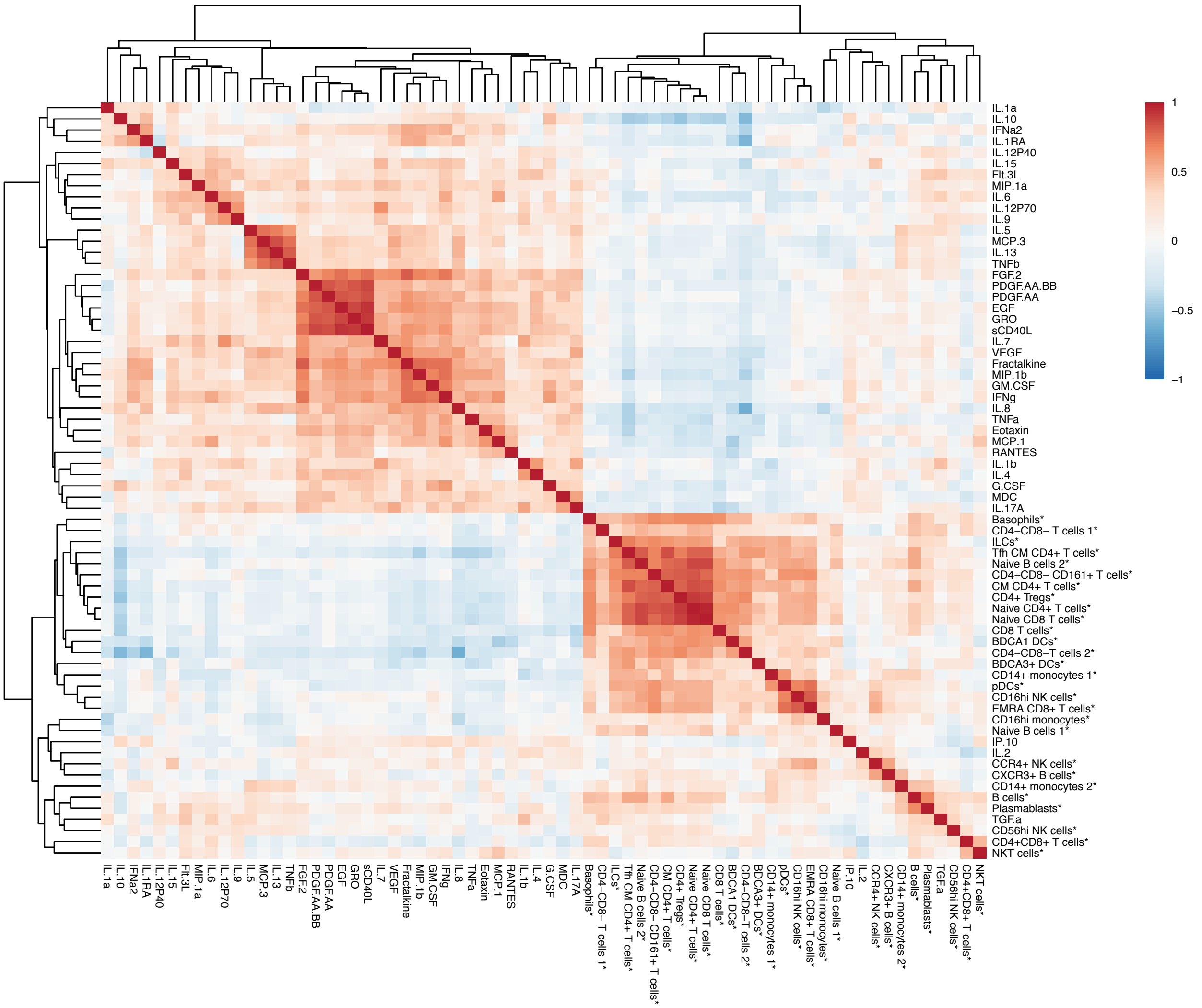

151

152 **Appendix Figure S13.**

153 Clustered heatmap of Spearman correlations between log-scaled serum cytokine concentration

154 (Luminex) and log-scaled cell subphenotype frequencies (CyTOF) *of changes from the acute to*

155 *convalescent timepoints*. CyTOF values are indicated by an asterisk following the label.

156

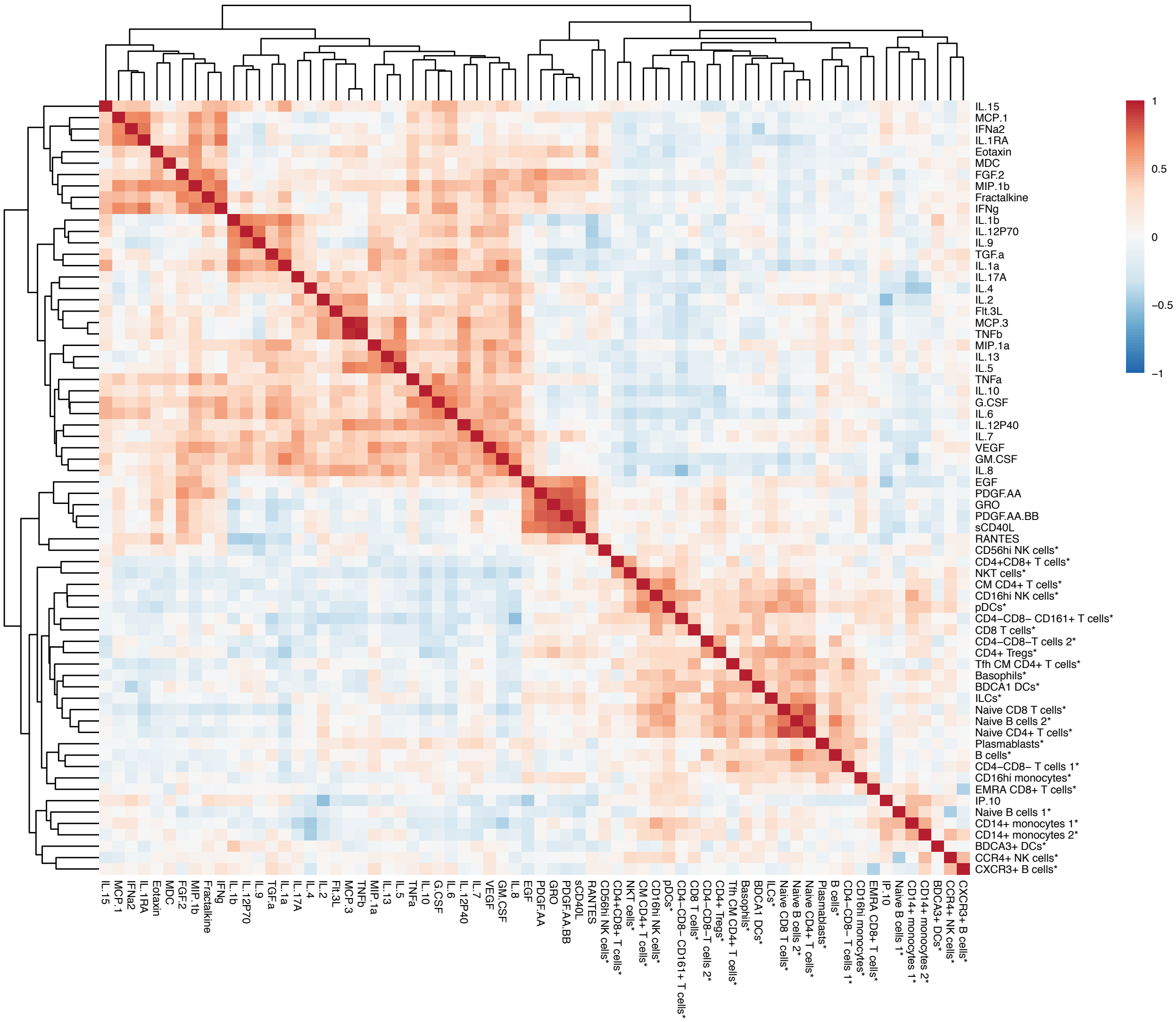

Appendix Figure S14.

Clustered heatmap of Spearman correlations between log-scaled serum cytokine concentration (Luminex) and log-scaled cell subphenotype frequencies (CyTOF) *within the acute timepoint*. CyTOF values are indicated by an asterisk following the label.

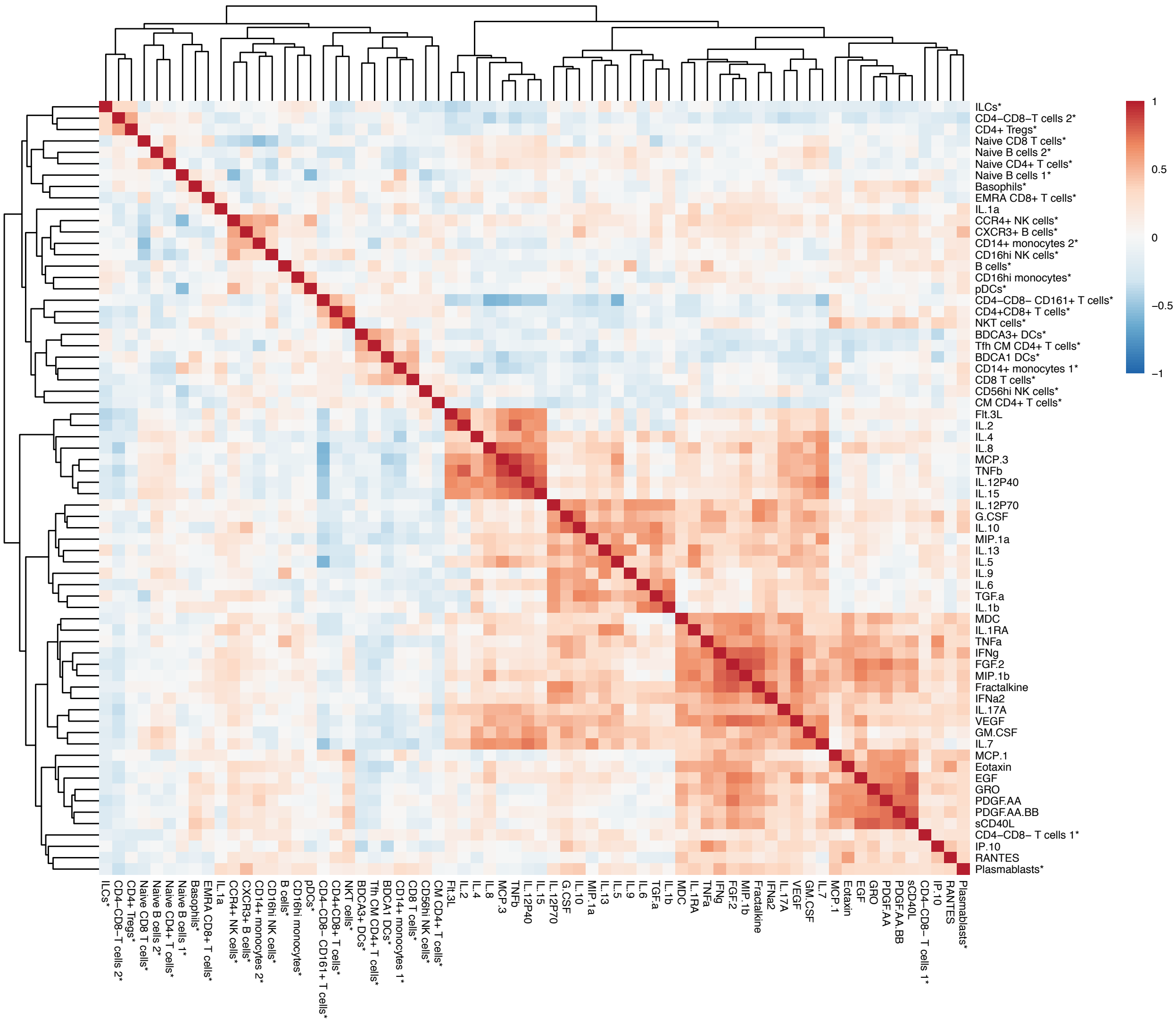

Appendix Figure S15.

Clustered heatmap of Spearman correlations between log-scaled serum cytokine concentration (Luminex) and log-scaled cell subphenotype frequencies (CyTOF) *within the convalescent timepoint*. CyTOF values are indicated with an asterisk following the label.

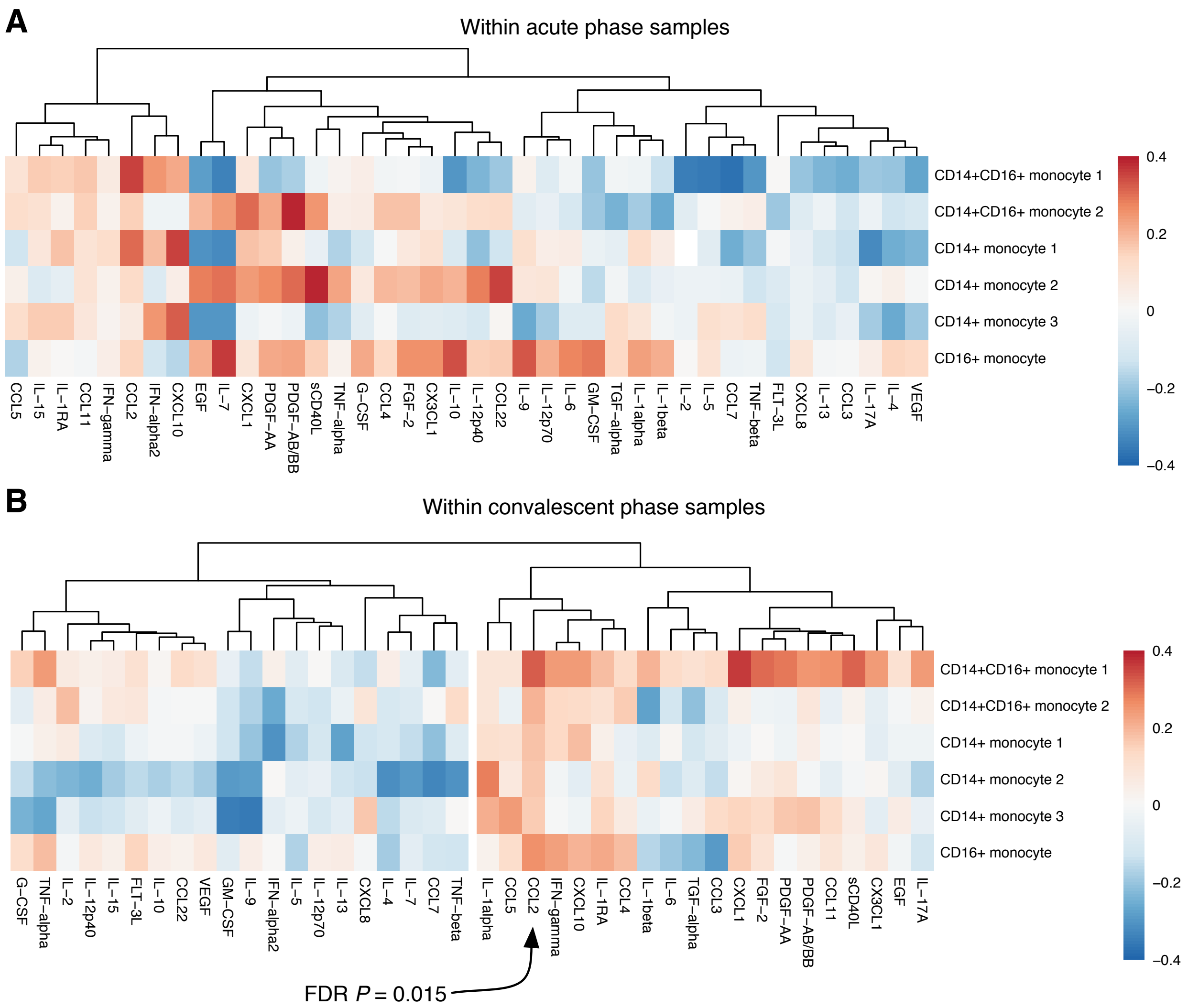

Appendix Figure S16.

Clustered heatmap of Spearman correlations between log-scaled serum cytokine concentration and log-scaled monocyte subphenotype frequencies. *A*, within *acute*-phase samples. *B*, within *convalescent*-phase samples. CCL2 within the convalescent timepoint (highlighted) displayed the only set of Spearman correlations that differed significantly from those of the other cytokines at an FDR threshold of < 0.05 (Mann-Whitney *U* test).

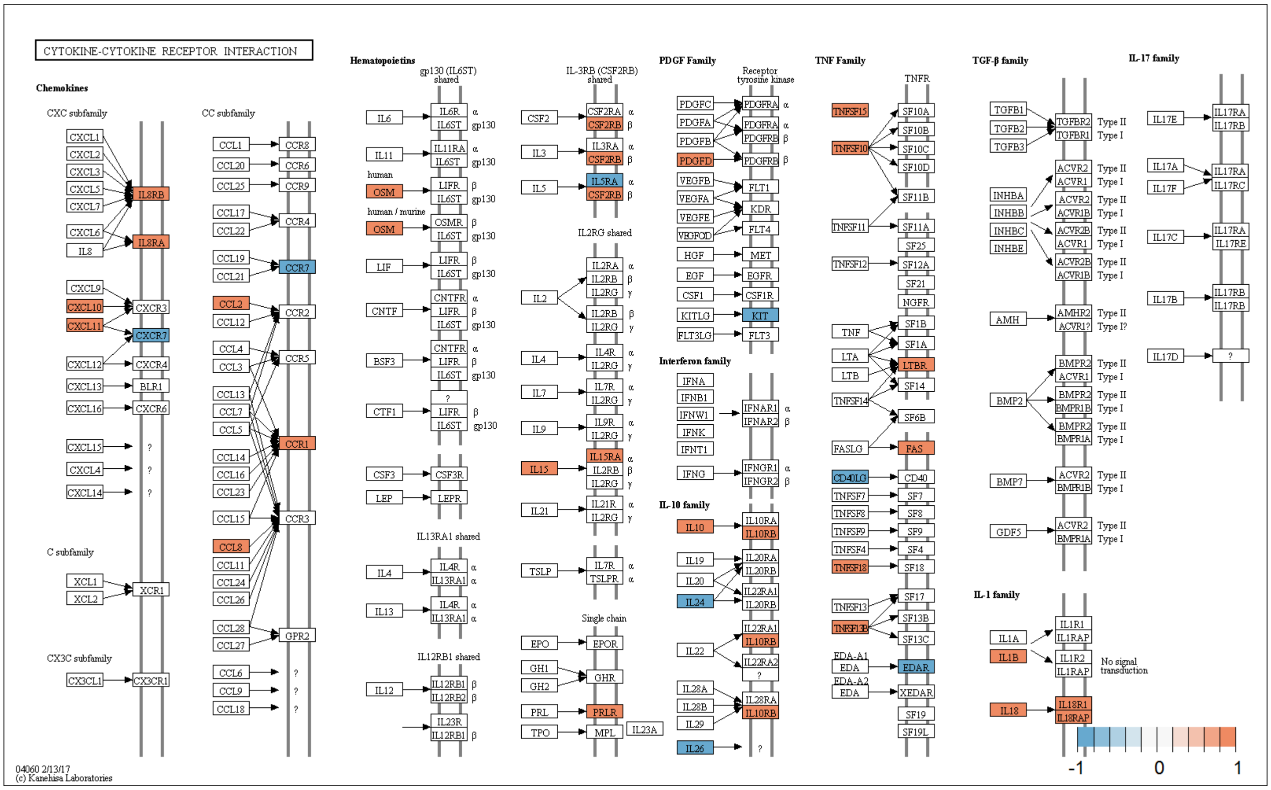

Appendix Figure S17.

Pathview plot of $\log_2$ fold change in gene expression between acute and convalescent timepoints for the cytokine-cytokine receptor interaction pathway in KEGG (accession hsa04060). Positive values indicate upregulation during the acute phase of infection.

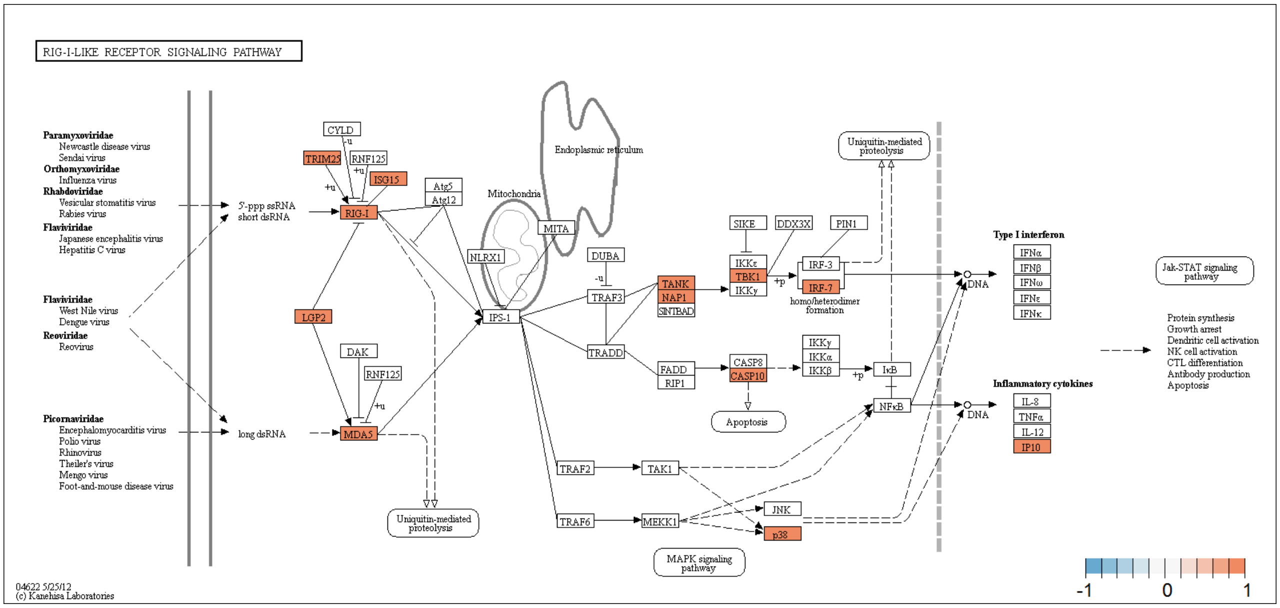

Appendix Figure S18.

Pathview plot of $\log_2$ fold change in gene expression between acute and convalescent timepoints for the RIG-I-like receptor signaling pathway in KEGG (accession hsa04622). Positive values indicate upregulation during the acute phase of infection.

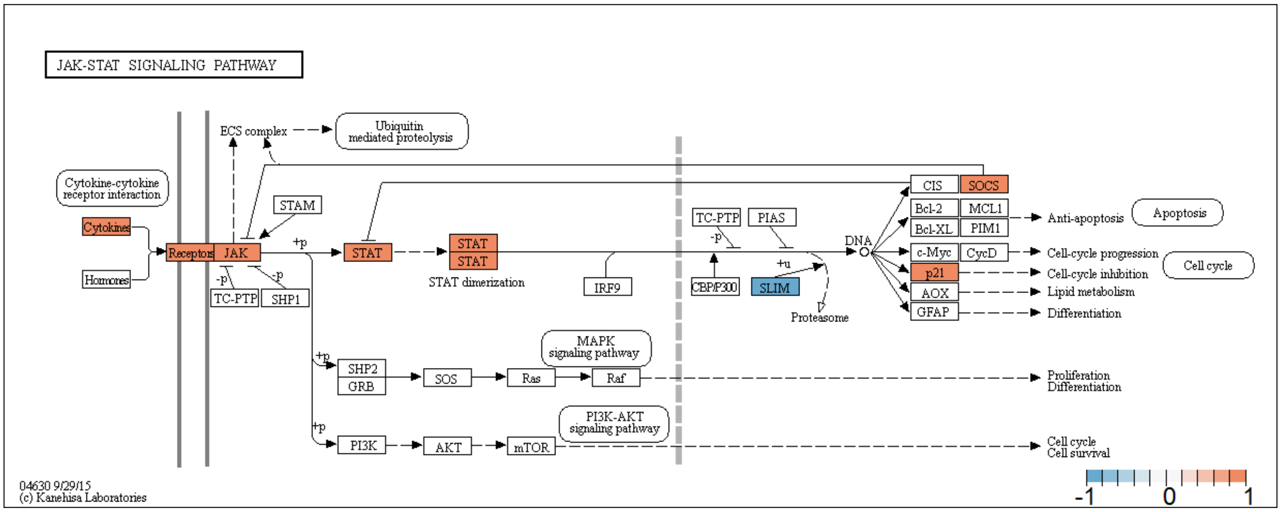

Appendix Figure S19.

Pathview plot of log_2 fold change in gene expression between acute and convalescent timepoints for the JAK-STAT signaling pathway in KEGG (accession hsa04630). Positive values indicate upregulation during the acute phase of infection.

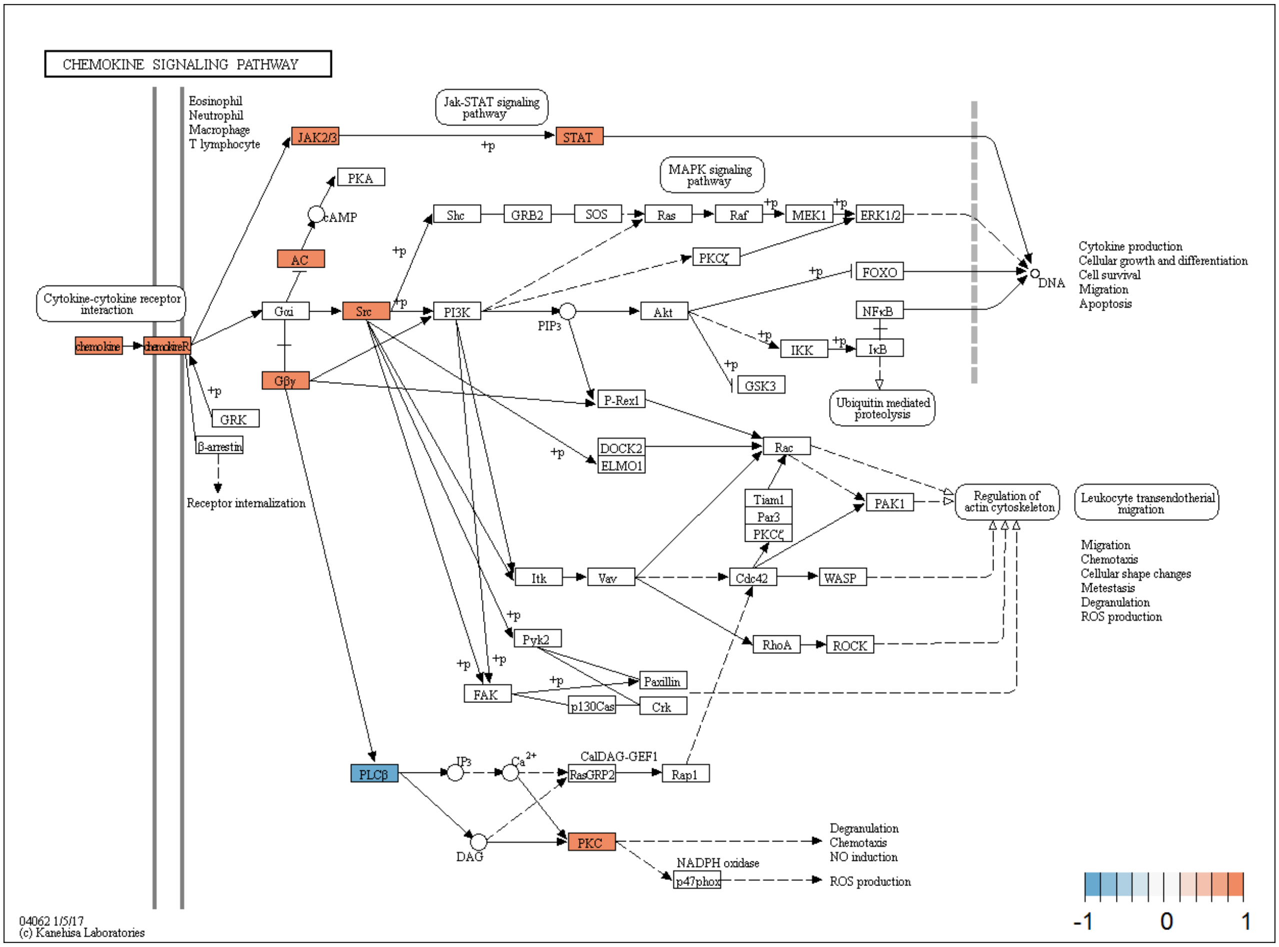

Appendix Figure S20.

Pathview plot of log_2 fold change in gene expression between acute and convalescent timepoints for the chemokine signaling pathway in KEGG (accession hsa04062). Positive values indicate upregulation during the acute phase of infection.

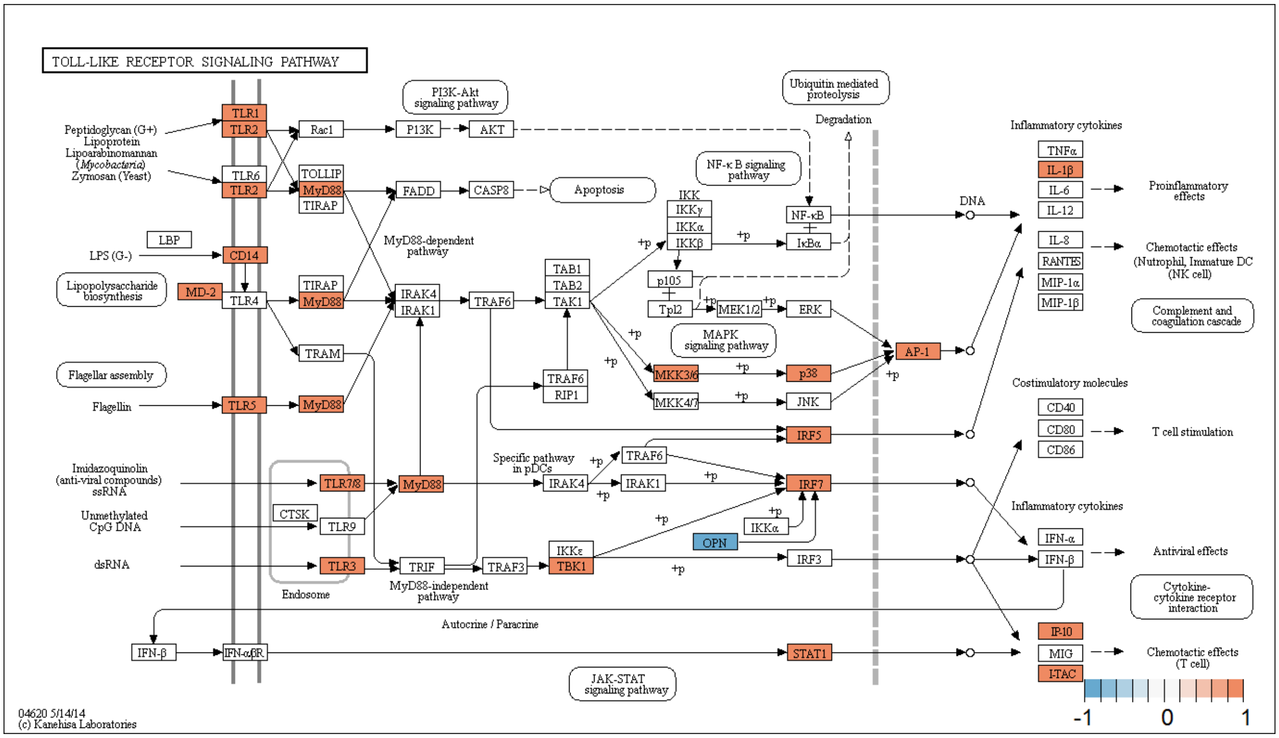

Appendix Figure S21.

Pathview plot of log_2 fold change in gene expression between acute and convalescent timepoints for the toll-like receptor pathway in KEGG (accession hsa04620). Positive values indicate upregulation during the acute phase of infection.

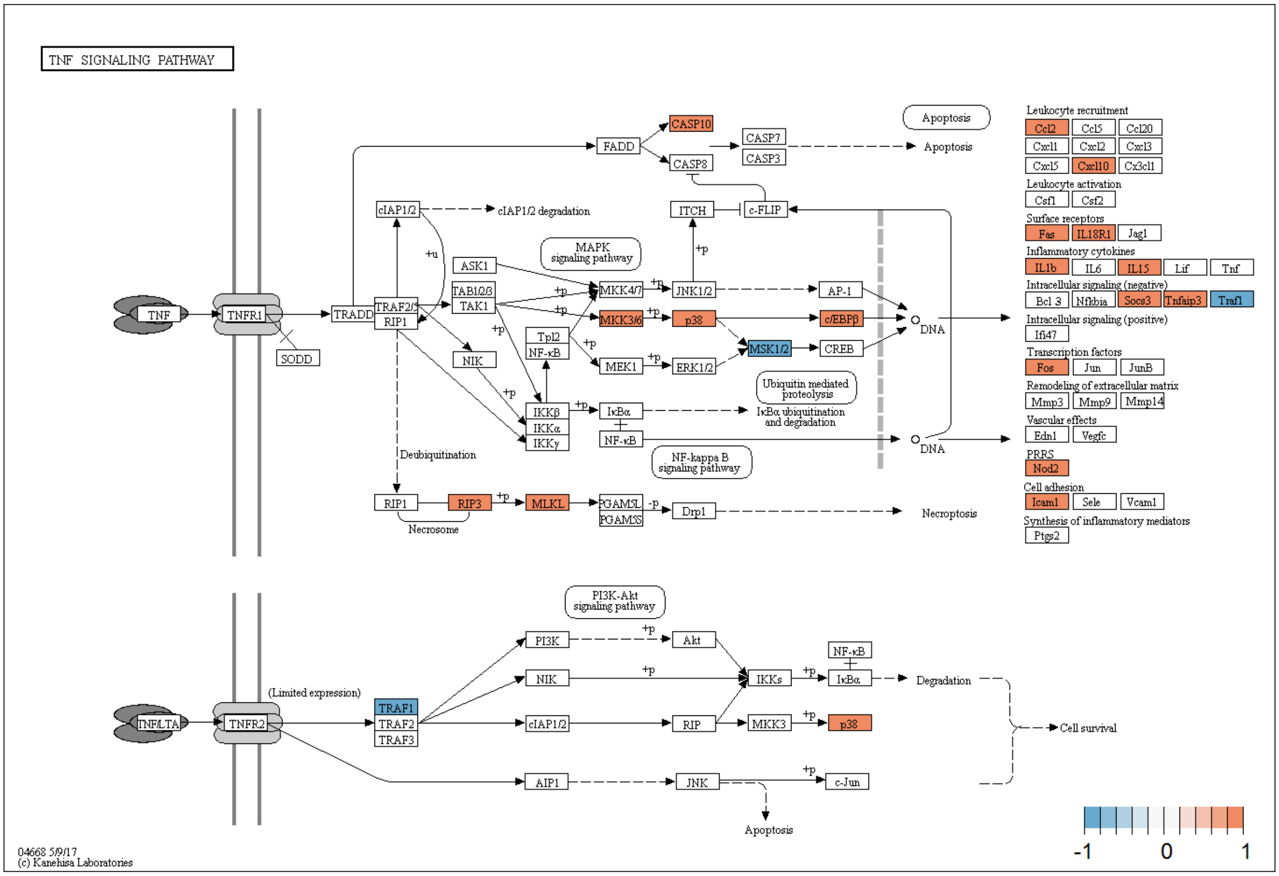

Appendix Figure S22.

Pathview plot of $\log_2$ fold change in gene expression between acute and convalescent timepoints for the TNF signaling pathway in KEGG (accession hsa04668). Positive values indicate upregulation during the acute phase of infection.

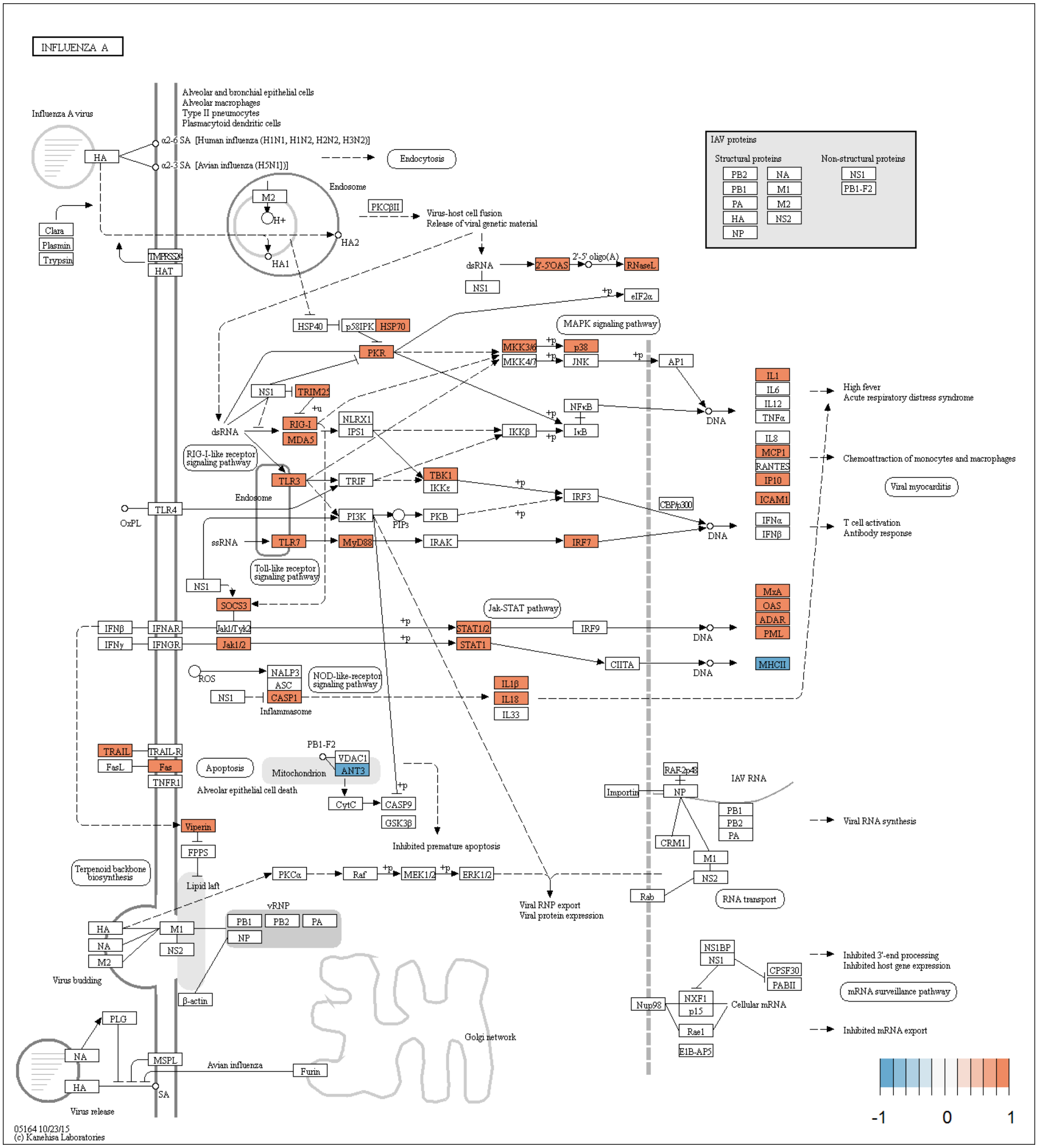

Appendix Figure S23.

Pathview plot of log_2 fold change in gene expression between acute and convalescent timepoints for the Influenza A pathway in KEGG (hsa05164). Positive values indicate upregulation during the acute phase of infection.

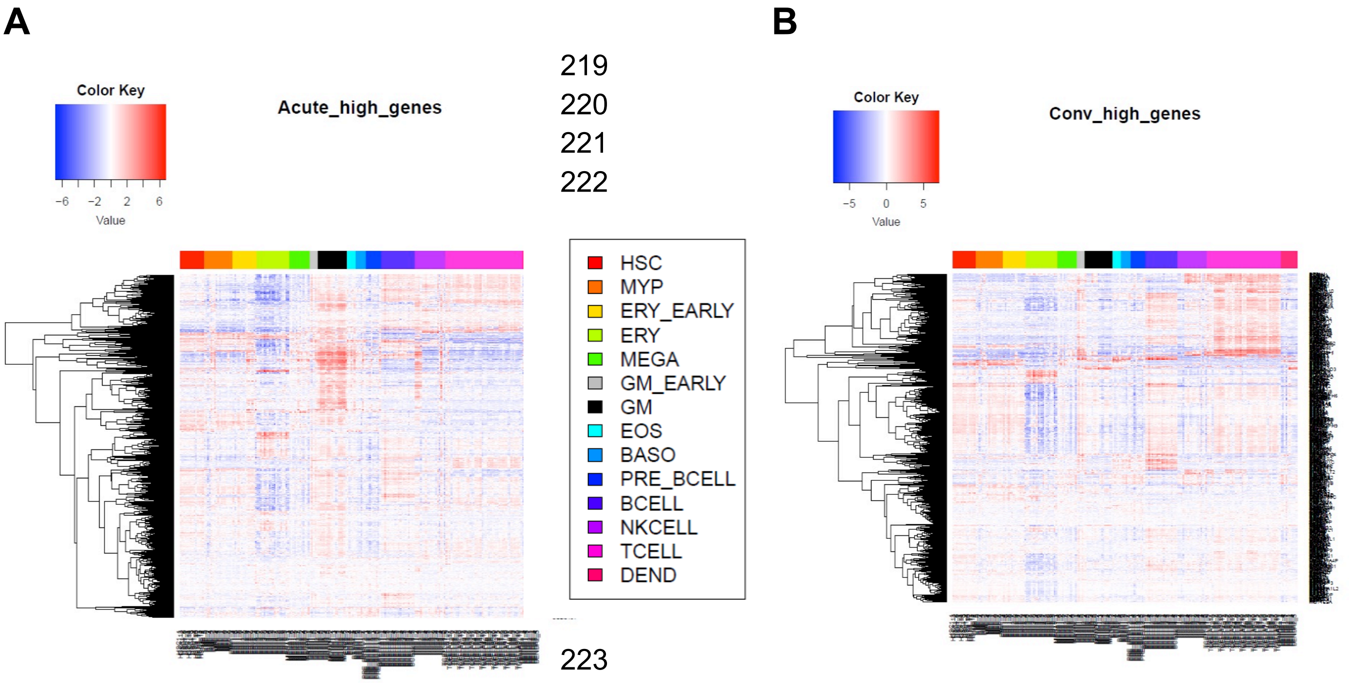

Appendix Figure S24.

Heatmap of gene expression across different types of blood cell lines for genes expressed higher in acute phase (*A*) or higher in convalescent phase (*B*). Rows represent genes. Columns represent blood cell lines which are grouped according to the lineage (column legend). HSC, Hematopoietic stem cell; MYP, Myeloid progenitor; ERY, Erythroid cell; MEGA, Megakaryocyte; GM, Granulocyte/monocyte; EOS, Eosinophil, BASO, Basophil; DEND, Dendritic cell.

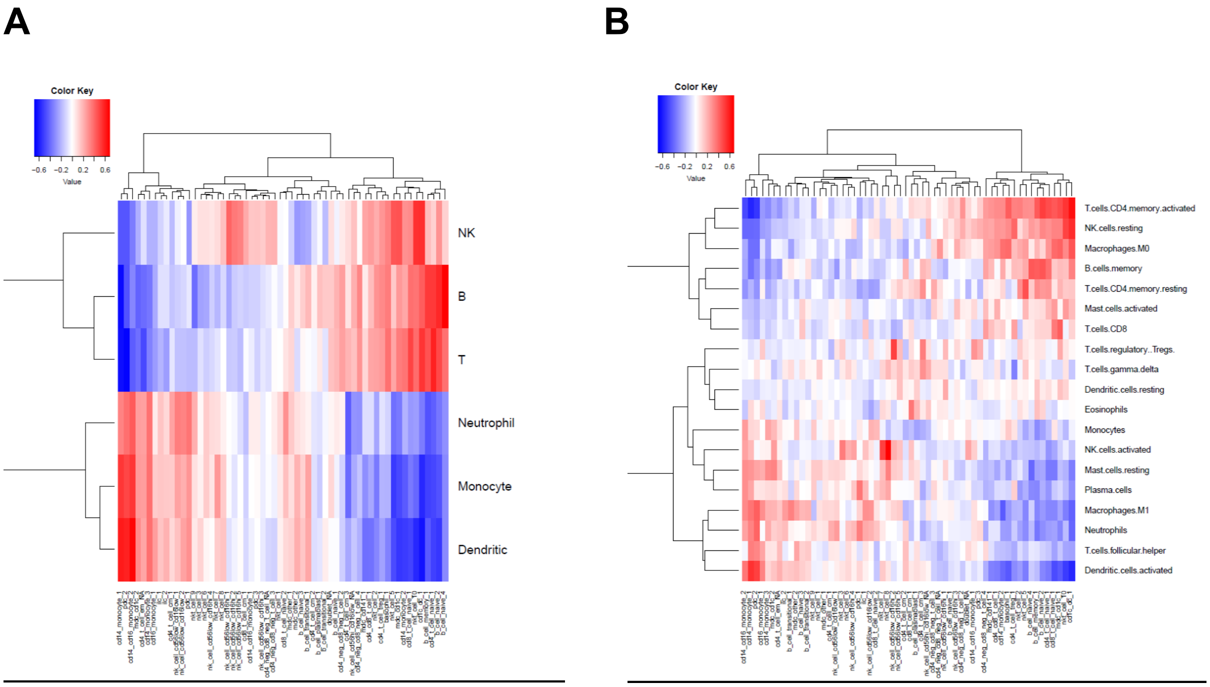

Appendix Figure S25.

Correlation between cell frequency derived from CyTOF and that estimated from gene expression using expression of cell-specific markers (*A*) or the CIBERSORT algorithm (*B*).

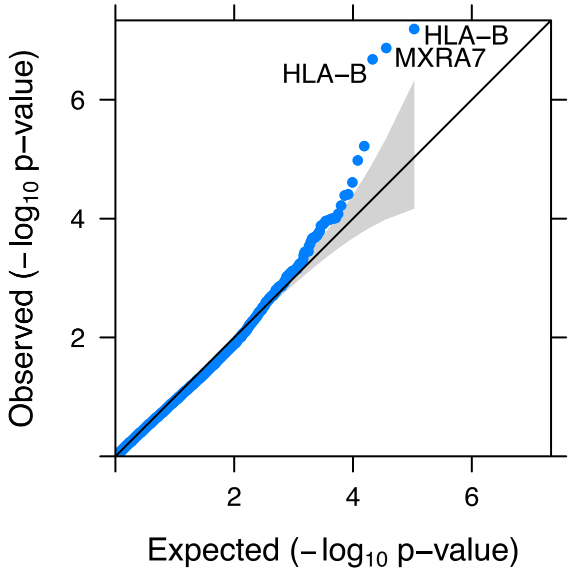

Appendix Figure S26.

Q-Q plot of the distribution of observed –$\log_{10}$ *P* values for differentially expressed transcripts between severe and non-severe cases against the distribution of –$\log_{10}$ *P* values expected under the null hypothesis (that no transcripts are differentially expressed). Gray shaded band indicates the 95% confidence interval for the expected *P* value distribution under the null hypothesis.

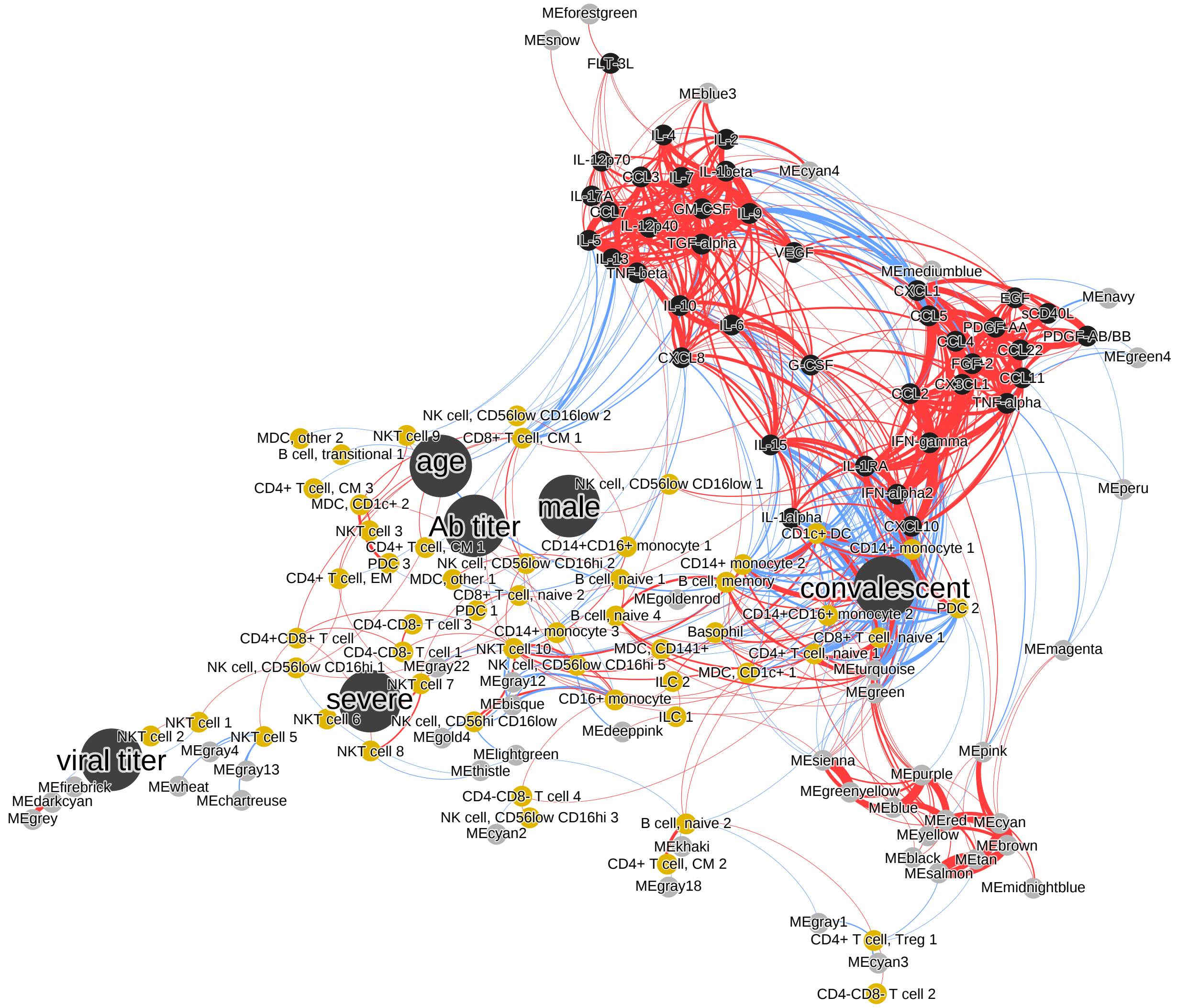

Appendix Figure S27.

Weighted multiscale interaction network depicting all Pearson's correlations between subpopulation frequencies (gold nodes), serum cytokine concentrations (small black nodes), coexpression modules (ME prefix, gray nodes), and clinical variables (large black nodes). Serum cytokine concentrations cluster into two highly interconnected components (upper right), farther from the clinical variables than the other node types. Edges are filtered to correlations significant at $P < 0.001$, and thickness is scaled to the magnitude of the correlation.

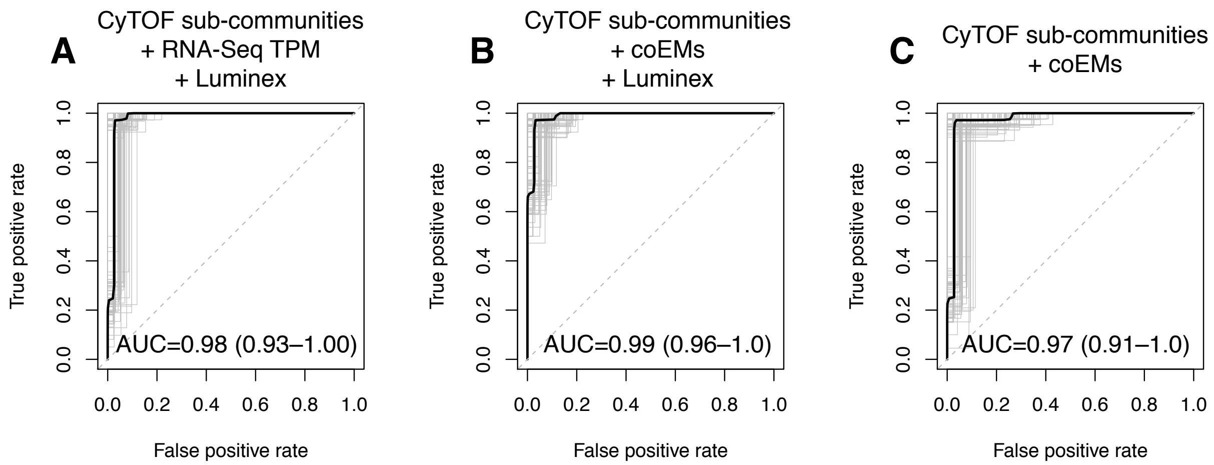

Appendix Figure S28.

Receiver operator characteristic (ROC) curves measuring the performance of elastic net logistic regression models predicting the phase of infection (acute vs. convalescent) for each sample, using progressively dimensionality-reduced versions of the dataset. Thin grey lines show the ROC curves for 100 bootstrap replicates. The area under the curve (AUC) along with its 95% confidence interval are shown underneath each plot; a perfect classifier would achieve AUC=1 while a random classifier is expected to achieve AUC=0.5 (dashed diagonal line). *A*, model trained using all CyTOF sub-community frequencies, all quantified RNA-seq transcripts, and all Luminex cytokine measurements achieves near-perfect performance. *B*, model trained as in *A* but with eigengene values for 92 coexpression modules replacing the RNA-seq transcript-level quantification; performance is still near-perfect. *C*, model trained as in *B* but without the Luminex cytokine measurements; performance is still essentially equivalent to the other two models.

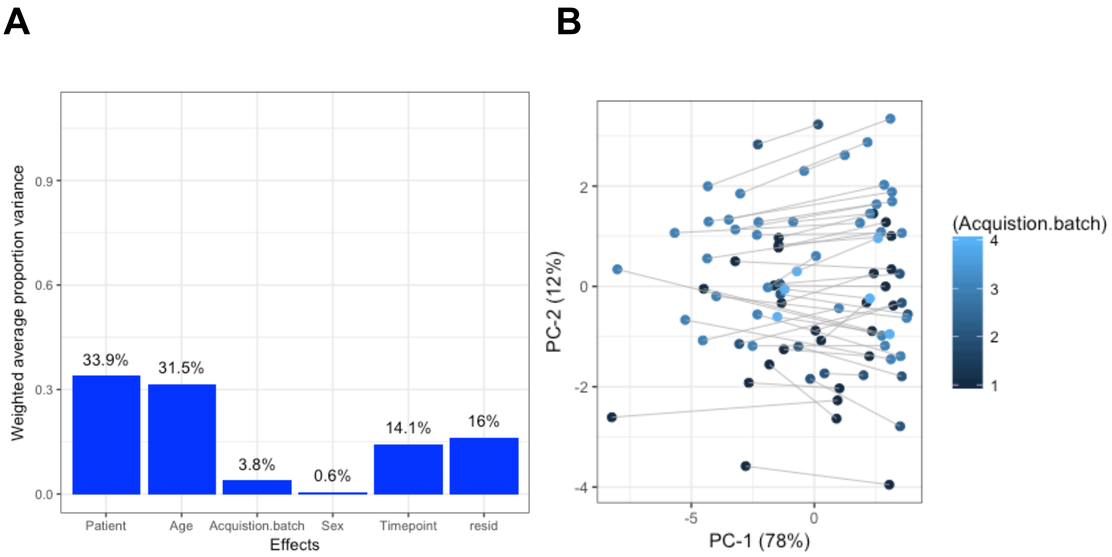

Appendix Figure S29.

Principal variance component analysis of CyTOF data for evaluation of potential batch effect. Since the 3 potential batch effect variables were largely collinear (Spearman correlation > 0.93 for thaw, staining and acquisition batch variables), we included only the acquisition date in our PVCA analysis. *A*, Barplot of contributions for each variable to overall variance. Acquisition date explains 3.8% of the overall variance. *B*, Scatterplot of all samples in principal component space for the first two principal components. Acquisition date is used to color the samples, and grey lines connect pairs of samples across the two timepoints. As expected, the first principal component (explaining 78% of the overall variance) roughly parallels the timepoint contrast, while the acquisition date variable does not correlate with either of the first two principal components.

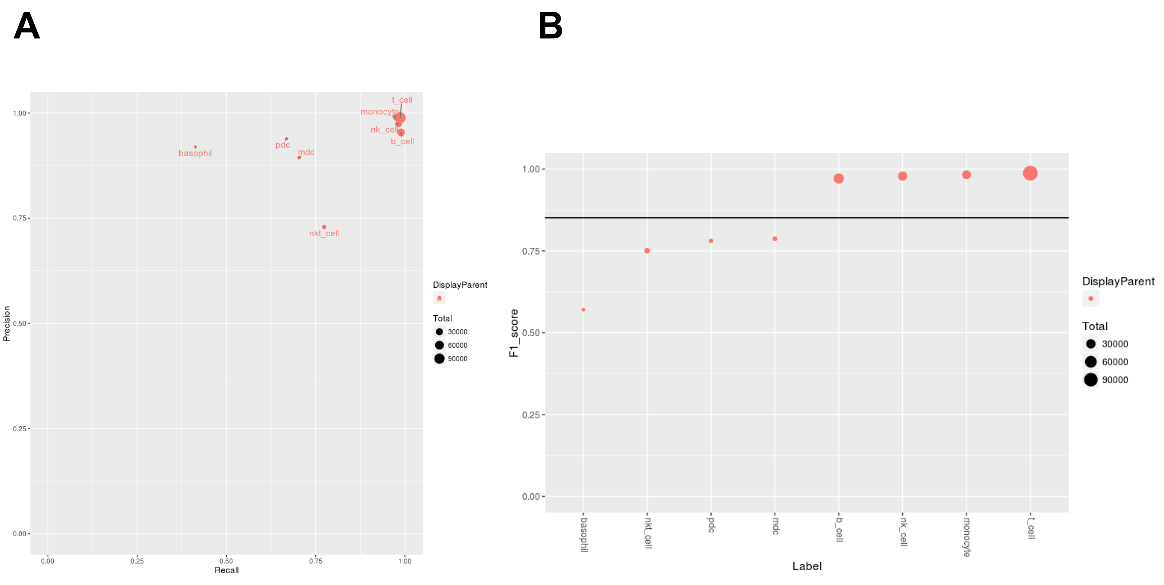

Appendix Figure S30.

High precision, recall scores and F1 score show that the classifier is returning accurate results with low false negative rates. *A,* Precision versus recall for each cell subset. The NOD classifier was trained over all samples except one, then applied to the remaining sample. This process was repeated for all samples ("jackknifing"). For each cell subset, precision (TP / TP + FP) and recall (TP / TP + FN) values were calculated, and the mean over all samples is presented here. *B,* The classifier is an accurate classifier as shown by high F1 score values for all large cell subsets. The F1 score for each cell subset is shown. The F1 score was calculated as the harmonic mean of precision and recall (2 x precision x recall / (precision + recall)). The line denotes the mean F1 score over all subsets. TP, true positive; FP, false positive; FN, false negative.
